# Fish Processing and Digestion Affect Parvalbumins Detectability in Gilthead Seabream and European Seabass

**DOI:** 10.3390/ani12213022

**Published:** 2022-11-03

**Authors:** Denise Schrama, Cláudia Raposo de Magalhães, Marco Cerqueira, Raquel Carrilho, Dominique Revets, Annette Kuehn, Sofia Engrola, Pedro M. Rodrigues

**Affiliations:** 1 Centre of Marine Sciences (CCMAR), Campus de Gambelas, Universidade do Algarve, 8005-139 Faro, Portugal; 2 Departamento de Ciências do Mar, da Terra e do Ambiente, Faculdade de Ciências e Tecnologia, Campus de Gambelas, Universidade do Algarve, 8005-139 Faro, Portugal; 3 Department of Infection and Immunity, Luxembourg Institute of Health, 29, Rue Henri Koch, L-4354 Esch-sur-Alzette, Luxembourg; 4 Departamento de Química e Farmácia, Faculdade de Ciências e Tecnologia, Campus de Gambelas, Universidade do Algarve, 8005-139 Faro, Portugal

**Keywords:** parvalbumin, gilthead seabream, European seabass, gastrointestinal digestion, fish processing

## Abstract

**Simple Summary:**

Fish provides high nutritional value in human diets but may trigger severe allergenic reactions, which result from a hypersensitive response of the immune system. Due to several allergenic proteins, and especially to parvalbumin, consumers with a known fish allergy must avoid any product that might contain this protein. This study focused on the characterization of parvalbumin in gilthead seabream and European seabass. Using mass spectrometry and circular dichroism, parvalbumins primary (sequence) and secondary structures were determined, respectively. Furthermore, parvalbumin was detected by sandwich enzyme-linked immunosorbent assay after gastrointestinal digestion and fish processing techniques. Parvalbumin—presented as α-helices and β-sheets, at room temperature—was detected at lower levels during gastrointestinal digestion. Several processing techniques showed a significant reduction (*p* < 0.05) in parvalbumin detectability, in comparison to raw muscle samples from gilthead seabream and European seabass. Therefore, we concluded that parvalbumins from both species are susceptible to digestion and processing. These results demonstrate that these techniques can be used in parvalbumin modulation and may be an important contribution to further studies on fish allergenicity.

**Abstract:**

Consumption of aquatic food, including fish, accounts for 17% of animal protein intake. However, fish consumption might also result in several side-effects such as sneezing, swelling and anaphylaxis in sensitized consumers. Fish allergy is an immune reaction to allergenic proteins in the fish muscle, for instance parvalbumin (PV), considered the major fish allergen. In this study, we characterize PV in two economically important fish species for southern European aquaculture, namely gilthead seabream and European seabass, to understand its stability during in vitro digestion and fish processing. This information is crucial for future studies on the allergenicity of processed fish products. PVs were extracted from fish muscles, identified by mass spectrometry (MS), and detected by sandwich enzyme-linked immunosorbent assay (ELISA) after simulated digestion and various food processing treatments. Secondary structures were determined by circular dichroism (CD) after purification by anion exchange and gel filtration chromatography. In both species, PVs presented as α-helical and β-sheet structures, at room temperature, were shown to unfold at boiling temperatures. In European seabass, PV detectability decreased during the simulated digestion and after 240 min (intestinal phase) no detection was observed, while steaming showed a decrease (*p* < 0.05) in PVs detectability in comparison to raw muscle samples, for both species. Additionally, freezing (−20 °C) for up to 12 months continued to reduce the detectability of PV in tested processing techniques. We concluded that PVs from both species are susceptible to digestion and processing techniques such as steaming and freezing. Our study obtained preliminary results for further research on the allergenic potential of PV after digestion and processing.

## 1. Introduction

Increasing fish consumption is a viable alternative to land-animal based diets due to its higher nutritional value. Easily digestible proteins of high quality, omega-3 fatty acids and vitamins are examples of the high nutritional value of fish [1]. Due to this knowledge, global demand for fish and fish products is increasing every year, with aquaculture playing an important role in this prospect [2]. In 2020, global fish production for human consumption reached 157 million tonnes, corresponding to 20.2 kg per capita/year [3]. Fish consumption contributed 17% of the intake of animal proteins globally [3]. Portugal has a long history of fish consumption and has the highest intake in the European Union (EU), with 59.91 kg/capita/year compared to an average of 23.97 kg/capita/year [4]. Gilthead seabream *Sparus aurata*, and European seabass *Dicentrarchus labrax*, used in this study, are examples of two economically important species highly consumed and produced in southern Europe.

Fish are among the most common food allergies worldwide and result from an excessive immune system response to specific proteins, called allergens [5]. Avoiding fish consumption might be a constraint in countries where high exposure to fish is common [6]. A better understanding of the allergenicity of specific fish species could assist fish-allergic consumers in selecting the best fish for consumption [7,8].

Fish parvalbumin (PV) is an allergen that is the main contributor to the clinical reactivity of fish-allergic patients. Thus, this allergen appears to be an important target to evaluate the allergenicity of fish species [9]. The highly stable Ca^2+^-binding PV is an EF-hand muscle protein with a molecular weight of 10–12 kDa [10]. Indeed, muscle PV regulates intracellular Ca^2+^-ion concentration (or Mg^2+^-ion), involved in the process of muscle relaxation [11], via two EF-hand domains [12]. Several studies on PV detection using IgG assays were performed with different fish species. PV from carp, catfish, cod and tilapia was positively identified using an anti-frog monoclonal antibody, but on the contrary, yellowfin tuna did not react [13]. Using specific PV antibodies for each fish species, salmon, carp, cod, mackerel, redfish, tuna and herring PV was identified by immunoblotting [14]. Studies on PV detection and epitopes in gilthead seabream, using mass spectrometry, were also previously reported [15,16]. It has been suggested that PV secondary structure suffers substantial changes upon calcium depletion, which seems to decrease its stability [17].

Evidence showed that PV stability decreases through its denaturation during specific digestion conditions or product processing. Allergen denaturation may lead to aggregates, which might change the way epitopes are recognized (being masked, un-masked or damaged) [18], and which may offer an accessible first step to desensitize consumers to have access to higher quality protein [19,20]. In this sense, fish allergen risk assessment is important to help consumers decide if a specific fish contains a known allergen and if it might trigger an allergic reaction [21,22], which is shaped by the method of detection. For instance, digestibility is one of the criteria used in fish allergy risk assessment evaluations where digested allergens are denatured or degraded. Denaturation and/or degradation of PV through the gastrointestinal tract depends on several factors, such as environmental pH and digestive enzymes [23,24]. Additionally, the pepsin to allergen ratio is crucial for the stability of PV [23]. However, extractability might change and partially or totally digested allergens could be in the insoluble fraction. Another important method to analyze fish allergen risks are processing methods (e.g., heating, pressure, salting and freezing) which are used for different purposes, such as improving fish quality (flavor, appearance) or extending shelf life [18]. Besides this, freezing is a convenient process for consumers. Additionally, processing techniques, such as thermal and non-thermal approaches, may change PV stability through conformational changes in structure (e.g., by destroying functional epitopes) [25]. A study on cod PV stability using heat and pressure showed protein denaturation with a pressure–temperature phase diagram [26]. In the case of the non-thermal salting process of herring, protein denaturation was also observed by SDS-PAGE [27].

This work, therefore, aimed to evaluate PV stability upon exposure to in vitro simulation of the gastrointestinal tract and different fish processing techniques (i.e., heating, pressure, salting and freezing) using an in-house developed sandwich ELISA (specific for gilthead seabream and European seabass). PV conformation in native and calcium-depleted apo-form was determined by circular dichroism. With this approach we expect to lay the molecular foundation for future research on the allergenic potential of processed fish products.

## 2. Material and Methods

### 2.1. Fish and Ethical Legislation

Gilthead seabream (*Sparus aurata)* and European seabass *(Dicentrarchus labrax*) juveniles were sampled (after being lethally anaesthetized with MS-222 (Merck)), from rearing tanks at the Ramalhete field station (CCMAR/University of Algarve, Portugal). Research on these species reared in the same infrastructure was previously published by our group in the context of fish allergenicity [28,29,30]. Fillets of 6 fish and dorsal muscle samples from another three were taken and frozen at −20 °C or −80 °C, respectively for both species. Fish skin was removed in either species before freezing. The experiments in this project were approved by the Portuguese National Authority for the Animal health (DGAV) with permit no. 003894, following guidelines for fish welfare established in Council Directive 2010/63/EU and Portuguese legislation for the use of laboratory animals, permit number 0420/00/000-n.9909/11/2009.

### 2.2. Protein Extraction

Proteins were extracted from the dorsal muscle samples of each species (three biological replicates) using a Retsch Mixer Mill MM 400 (Retsch, Haan, Germany) tissue laser as described by Kalic, et al. [31] with some slight modifications. Briefly, one hundred mg of each muscle tissue were added to 1 steel bead of 5 mm in diameter and 1 mL of detergent lysis buffer (50 mM Tris-HCl, pH 8.0, 150 mM NaCl, 1% Triton X-100) as extraction buffer. Samples were homogenized for 10 min at 25 Hz, and incubated on an orbital shaker for 1 h, at 4 °C, to homogenize the suspension. Following, samples were centrifuged at 20,000× *g* for 15 min at 4 °C. The protein content on the collected supernatant (crude extract) was quantified using the Bradford assay with bovine serum albumin (BSA) as standard protein.

### 2.3. Parvalbumin Purification

As a pre-separation step, crude extracts from both species were heated at 90 °C for 10 min, followed by centrifugation at 20,000× *g* for 15 min [31,32]. The collected supernatant was quantified using the Bradford assay with bovine serum albumin (BSA) as standard protein. To check the presence of other potential heat-resistant proteins besides PV, 10 μg of protein of each sample were loaded and separated by SDS-PAGE, using the AnykD™ Mini-Protean^®^ TGX gels (Bio-Rad Laboratories, Hercules, CA, USA), for 35 min at 200 V followed by Coomassie blue staining. PV was identified by mass spectrometry and IgG-recognition using commercial anti-PV antibodies (see Supplementary Materials—S1).

To purify PV from a sample mixture [14], the three heated extracts per fish species were pooled. Prior to chromatography, both samples were dialyzed against 20 mM Tris, pH 8 (Amicon^®^ Ultra-15; Merck Millipore Ltd., Cork, Ireland). Proteins were separated by anion exchange chromatography (Resource™ Q; GE Healthcare, Uppsala, Sweden), on an NGC instrument (Bio-Rad Laboratories, Hercules, CA, USA) using an elution gradient of 0–1 M NaCl in 20 mM Tris (pH 8). The flow rate was set at 0.5 mL/min and the UV detector at a wavelength of 280 nm. PV-containing fractions were pooled, concentrated (Amicon^®^ Ultra-15; Merck Millipore Ltd., Cork, Ireland) and loaded on a high-performance gel filtration column (Superdex™ 75 10/300 GL, GE Healthcare, Uppsala, Sweden) using 50 mM KH_2_PO_4_, 150 mM NaCl, pH 7, as running buffer. The flow rate was set at 0.5 mL/min and the UV detector at a wavelength of 280 nm. Purity of PV-containing fractions was verified by SDS-PAGE followed by SYPRO™ Ruby staining (Thermo Fisher Scientific, Waltham, MA, USA, see Supplementary Materials—S2). Further on in the manuscript, when referring to purified proteins, these are the ones obtained after gel filtration chromatography.

### 2.4. Identification of Parvalbumin by Mass Spectrometry

PVs were identified by mass spectrometry (MS) analysis. Bands were manually excised from SDS-PAGE gels from heated extracts, reduced with dithiotreitol (DTT) (10 mM, for 20 min, at 55 °C) and digested with trypsin (12.5 ng/μL, 50 mM NH_4_HCO_3_, pH 8) for 90 min at 37 °C (TrypsinGold, Promega, Madison, WI, USA). Finally, 0.5 μL of the digested sample and 0.3 μL of the matrix solution (25 mg/mL α-cyano-4-hydroxycinnamic acid and 4 mg/mL 2,5-dihydroxybenoiquem in 50% acetonitrile containing 0.1% TFA) were spotted on a MALDI-plate (Polished steel 384 MALDI target plate, Bruker, Germany). A protein mass fingerprint (PMF) analysis was performed on the MALDI-TOF/TOF MS (Ultraflex I, Bruker Daltonics, Bremen, Germany). Calibration of the MALDI-TOF-MS was performed in two steps: the external one was performed by mass scale calibration using a tryptic digested BSA according to manufacturer’s instructions (Bruker Daltonics, Bremen, Germany). In brief, a reference mass list of ionized peptides from tryptic digested BSA (calculated masses) was compared with the list of measured masses. A polynomial function was applied to the measured mass list to match with the reference mass list as closely as possible. This first calibration was a pre-acquisition calibration. The internal calibration was performed by the tryptic peptides coming from the auto digestion of the enzyme. They were used as a second calibration for the protein mass fingerprint analysis to optimize the calibration before each request into the database. This second calibration was a post-acquisition calibration. After conversion to MS and MS/MS peak lists, the search was restricted to the Actinopterygii database (NCBI: txid7898) with 100 ppm of mass error tolerance in MS and MS/MS precursor, and 0.3 Da tolerance on MS/MS fragments.

### 2.5. Biomolecular Characterization by Circular Dichroism

After chromatography, purified PVs were used for biomolecular characterization. Secondary protein structures of European seabass and gilthead seabream PVs were compared by circular dichroism (CD) spectroscopy [33]. First, spectra were recorded at 20 °C using a Chirascan CD spectrometer (Applied Photophysics, Leatherhead, UK). Afterwards, a temperature ramping from 20 °C to 95 °C was performed (with a ramping rate of 0.82 °C per minute). After 10 min at 95 °C, a subsequent scan was taken, and samples were tempered back to 20 °C for a final scan to analyze the refolding capacity. All measurements were performed with a 0.1 cm optical path length quartz cell to obtain spectra in the far-UV region (180 to 260 nm) at a protein concentration of 0.2 μg/μL in 10 mM KH_2_PO_4_ pH 7. The CD spectra were acquired at a scan speed of 0.4 nm/s and a step resolution of 0.7 nm. Spectra were measured in five analytical replicates and averaged. PVs stability can be explained by its structure, which depends on calcium chelating. Upon depletion of calcium, conformation changes in PV might result in lower IgE reactivity. Therefore, all samples were analyzed in native conditions and after calcium depletion with 5 mM EDTA to determine the structure of PV in its apo-form (using the same conditions and settings as stated before). All spectra were blank subtracted, averaged and smoothed with a Savitzky–Golay filter (window size 5). Results were expressed in mean residue ellipticity (Θ mdeg) at a given wavelength. Secondary structure was determined using DichroWeb using reference data set 4 as established in [34].

### 2.6. In Vitro Simulation of the Gastrointestinal Tract

PVs resistance was analyzed by food digestion simulating the gastrointestinal tract, as described by Minekus, et al. [35], Akkerdaas, et al. [36]. The digestion parameters were determined based on physiological data. Briefly, 5 mg of crude extract (described earlier) muscle protein (three biological replicates per species) were digested in simulated gastric fluid (SGF, 6.9 mM KCl, 0.9 mM KH_2_PO_4_, 25 mM NaHCO_3_, 47.2 mM NaCl, 0.1 mM MgCl_2_(H_2_O)_6_, 0.5 mM (NH_4_)_2_CO_3_, 15.6 mM HCl, 0.15 mM CaCl_2_(H_2_O)_2_, pH 3) with 1 U pepsin per μg of protein for 2 h. Samples (100 μL) were taken after 0 min (before adding pepsin), 1, 2, 5, 10, 20, 30, 60, 90 and 120 min. Digestion was interrupted with 10 μL of NaOH 1 M and mixed thoroughly. To continue the digestion process, simulated intestinal fluid (SIF, 6.8 mM KCl, 0.8 KH_2_PO_4,_ 85 mM NaHCO_3_, 38.4 mM NaCl, 0.33 mM MgCl_2_(H_2_O)_6_, 8.4 mM HCl, 0.6 CaCl_2_(H_2_O)_2_, pH 7) was added to the remaining mixture. Pancreatin (800 U/mL) and bile salts (10 mM) were also added, and samples were taken at the same time points as stated above (digestion was interrupted by placing samples on ice). All steps were performed at 37 °C with 500 rpm on a ThermoMixer^®^ (Eppendorf, Hamburg, Germany). PV detectability was assessed by sandwich ELISA.

### 2.7. Sandwich ELISA

For PV detection, a new sandwich ELISA was produced in our laboratory and optimized using PV antibodies from purified protein obtained from European seabass and gilthead seabream. Purified antibodies were produced in rabbits (Supplementary Materials SMM1), immunized with the PV from both species previously purified by chromatography (Eurogentec, Seraing, Belgium). This approach achieved a high sensitivity for the detection of our native and processed fish samples [14]. Purified antibodies were further biotinylated using the EZ-link™ Sulfo-NHS-LC-Biotinylation kit following manufacturer’s instructions (Thermo Fisher Scientific, Waltham, MA, USA). ELISA plates (F96 Maxisorp immunoplate, Thermo Fisher Scientific, Waltham, MA, USA) were coated with anti-PV at 500 ng/well overnight at 4 °C as described by Kuehn, Scheuermann, Hilger and Hentges [14], Kuehn, et al. [37]. Wells were blocked with 300 μL of blocking solution (3% BSA in TBS-0.05% Tween (TBS-T)) for 4 h at room temperature (RT). Samples were analyzed in duplicate. A standard curve of purified PV, from each species, was added at 1000, 200, 100, 20, 4, 2, 0.4, 0.08 ng/mL. Wells were incubated with biotinylated anti-PV (1:2000) overnight at 4 °C, which was followed by streptavidin-alkaline phosphate (AP, Merck Millipore, Novagen, Darmstadt, Germany 1:1000) for 30 min at RT. For detection at 405 nm at a multiplate reader (BioTek Instruments, Winooski, VT, USA) alkaline phosphatase substrate solution was added to each well (p-Nitrophenyl Phosphate, pNPP, Merck) and continuous reading for 1 h with 5 min interval was performed. In between each step wells were washed with TBS-T for 5 times. Negative control was performed without adding sample to the well. LOQ and LOD were 0.06 and 0.02 ng/mL, respectively. Sample concentrations were calculated after plotting the standard values with a 4-parameter logistic curve fit using AssayFitPro for Excel.

### 2.8. Fish Muscle Processing and Conservation

Raw fish fillets of six animals each, gilthead seabream and European seabass, were frozen at −20 °C for 12 months to analyze the effects of freezing on the detectability of PV by sandwich ELISA. Three time points (0, 6 and 12 months) were chosen to extract proteins from fish muscle and to quantify PV by ELISA (described earlier). Raw muscle, at each time point, was previously processed with one of the following methods: no processing, raw (A), boiled with tap water at 95 °C in a water bath for 10 min (B), autoclaved at 121 °C for 30 min (C), salted with 5% NaCl (salt is used to provide flavor and when used for longer periods as preservation [38]) followed by autoclaving at 121 °C for 30 min (D) and steamed at 98–100 °C for 8 min followed by autoclaving at 121 °C for 30 min (E). Following, protein extracts were prepared from processed fish, including raw samples.

### 2.9. Statistical Analyses

Statistical analyses were performed using R v4.1.2 (R Core Team, 2020) for MacOSX. Data from digestion and processing techniques were transformed by log_10_ prior to statistical analysis. Significant differences among digestion samples were assessed by a one-way repeated measures analysis of variance (ANOVA), after verifying residual’s normality and homoscedasticity through Shapiro–Wilk and Levene’s test, respectively. Pairwise comparisons were assessed by a *t*-test with a Bonferroni correction as post hoc analysis. In case of a non-normal distribution a one-way repeated measures ANOVA of aligned rank transformed data was performed. This non-parametric test has been chosen over the Friedman test as it has more power and robustness when using small sample sizes [39]. Significant differences among processing samples were assessed by a two-way repeated measures ANOVA (a normal distribution was verified in all cases). Pairwise comparisons were assessed by a *t*-test with a Bonferroni correction as post hoc analysis. Significant differences were considered when *p* < 0.05.

## 3. Results and Discussion

PV, the main fish allergen, is known to be a stable protein against thermal and proteolytic degradation [40]. Nevertheless, it is also known that digestion conditions, such as low pH and proteolytic enzymes [41], and thermal and non-thermal processing might influence the structure (tertiary, secondary or primary) of PV [42] and consequently change its detectability. Hence, in this study we characterized the PV of two important southern Europe fish species by determining both its structure, through circular dichroism, and its stability, after exposure to gastrointestinal tract conditions and different processing techniques, using a sandwich ELISA. It should be noted that, using this ELISA, we could only detect the reaction of PVs epitopes to our antibodies, no conclusions could be drawn regarding the allergenicity.

### 3.1. Parvalbumin Purification

To visualize any protein resistant to this thermal process, protein extracts were heated at 90 °C for 10 min followed by SDS-PAGE. Subsequent PV detection with commercial antibodies was performed and shown in Supplementary Materials S1. In case of European seabass two clear bands appear at a low molecular weight, one closer to 14 kDa and another below 10 kDa. For gilthead seabream, two bands were visible after heating close to 10 kDa. The commercial antibodies (monoclonal anti-PV Swant PV235 and anti-rabbit/mouse IgG conjugated with AP, Sigma) detected both bands in European seabass, however the upper band showed a stronger reaction than the lower. For gilthead seabream, the positive bands were detected by the antibody. Therefore, we proceeded with the identification of these bands by MS and further purification steps by chromatography.

### 3.2. Parvalbumin Identification

**Figure 1:**
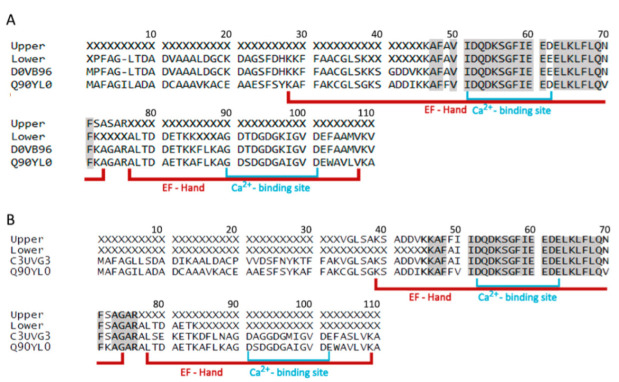
Parvalbumin sequences of gilthead seabream (**A**) and European seabass (**B**) obtained from positive bands presented in Figure S1, in comparison to the available sequence of gilthead seabream (Uniprot D0VB96), European seabass (C3UVG3) and cod parvalbumin (Q90YL0). Grey areas represent homology between sequences and X represents no MS identification. EF-Hand motifs and Ca^2+^-binding site are shown by red and blue lines, respectively.

Parvalbumin sequences of gilthead seabream and European seabass are available at the Uniprot database (uniprot.org). Identification, by MALDI-TOF/TOF MS, of the two obtained bands from the gilthead seabream muscle sample (after heating, Figure S1) showed 28.4% and 86.1% sequence coverage with PV from *Austrofundulus limnaeus* (Uniprot accession number A0A2I4CT95) and *Sparus aurata* (D0VB96_SPAAU), for the upper and lower band, respectively (Figure 1A). European seabass also showed two bands with low molecular weight, which were both identified as being PV from *Hypomesus transpacificus* (C3UVG3) and *Stegastes partitus* (A0A3B5AFY4) with sequence coverages of 39.4% and 35.8%, respectively (Figure 1B). When using the online available BLAST software (blast.ncbi.nlm.nih.gov/Blast.cgi) against the well-known cod PV (Q90YL0) sequence coverages of 87.1%, 63.6%, 86.1% and 92.3% were identified for the upper and lower band of gilthead seabream and European seabass, respectively. Sequence identities Ca^2+^-binding regions, known IgE-binding regions in codfish allergy, reached even up to 91.7% for gilthead seabream and 100% for European seabass, thus pointing to a possible basis for clinical cross-reactivity (Figure 1). It should be noted that for the blast search only part of the sequences was used as several amino acids were not identified by MS, resulting in higher coverages except for the lower band of gilthead seabream where the whole sequence was used.

### 3.3. Circular Dichroism

**Figure 2:**
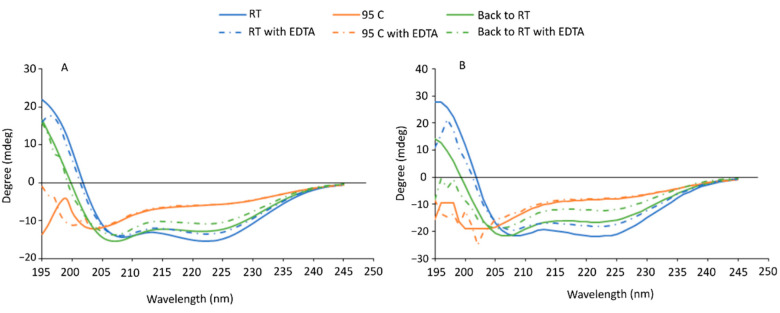
Far-UV circular dichroism analysis of purified β-parvalbumin of (**A**) European seabass and (**B**) gilthead seabream at RT (blue line), 95 °C (orange line) and back to RT (green line). Additionally, purified β-parvalbumin depleted with 5 mM EDTA (dashed lines) is shown for all conditions (RT, 95 °C and back to RT). *X*-axis represent wavelength (nm) and *y*-axis the measured ellipticity in degree (mdeg).

After PV identification and purification, we determined the secondary structure of the main fish allergen by using the spectroscopic method of circular dichroism (CD). β-PVs structure of European seabass and gilthead seabream, determined by far-ultraviolet CD, showed a similar profile for both species in both conditions (native and apo-form) (Figure 2A and Figure 2B, respectively). In case of the native structure (Figure 2, full lines) at room temperature (RT, 20 °C, blue lines), two minima were shown in the spectra, at 208 nm and 222 nm, and a maximum below 200 nm which is typical for proteins with some β-sheets and higher content of α-helices in its secondary structure (which was confirmed by DichroWeb) [43]. The monophasic unfolding melting point for European seabass and gilthead seabream was 69.9 ± 0.3 °C and 72.3 ± 0.1 °C, respectively, which are lower than the one determined in carp PV [44]. At 95 °C (orange lines, Figure 2), spectra showed a minimum at 204 nm and 200 nm, for European seabass and gilthead seabream, respectively, which is typical of proteins with a random conformation [45]. These results are in accordance with the ones determined for carp PV [44]. Cooling down back to RT (green lines, Figure 2) showed that PV from both species was almost able to refold back to its original structure (two minima were shown at 207 nm and 221 nm). Although we observed very similar minima and maxima wavelength values, comparing to RT structure, there was a slight shift in the zero-crossing (0 ellipticity) wavelength. We observed lower wavelengths at zero-crossing which might be due to loss of some α-helixes [43]. This was not observed in carp PV, which was able to refold its structure after heating [44].

Calcium chelator compounds such as ethylenediaminetetraacetic acid (EDTA), a safe and effective food additive to extend shelf life or as an emulsifier, are suggested to decrease PV stability. In case of the calcium-depleted structure (apo-form) (Figure 2, dashed lines) with 5 mM EDTA, a more notable difference was shown for the refolding capacity of gilthead seabream where a minimum of 205 nm and no stable maximum below 200 nm seems to influence slightly its structure. As such, the affected stability of PV might influence the binding to IgE and consequently modify its allergenic potential.

### 3.4. Parvalbumin Digestion

**Figure 3:**
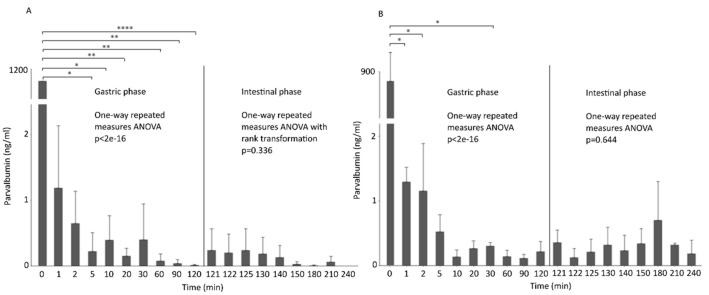
Bar plot of in vitro simulation of the gastrointestinal tract of muscle proteins (*n* = 3) from European seabass (**A**) and gilthead seabream (**B**). Parvalbumin was quantified using a sandwich ELISA with antibodies against the analyzed fish species. Asterisks indicate significant differences by paired *t*-test with Bonferroni correction (* *p* < 0.05, ** *p* < 0.01, **** *p* < 0.0001). *X*-axis represents time (min) and *y*-axis the measured parvalbumin (ng/mL).

To further characterize PV, we proceeded with an in vitro simulation of the gastrointestinal tract to evaluate the resistance of PV under static pH conditions, with pepsin activity in the gastric phase, and with bile salts and pancreatin present in the intestinal environment [46,47]. Yet, it is known that protein denaturation and degradation depends on gastric acidity and on the consequent proteolytic activity of pepsins [48]. Besides this, in vivo, a change from fasted to a fed state shifts the pH from 1 to 6 and higher, respectively. As explained previously, the detectability of PV was performed using a sandwich ELISA with specific antibodies produced for the fish species being studied. Using this detection model, we were able to understand how the PV epitopes change through digestion. Our in vitro digestion analysis, under static conditions in the gastric phase, showed in both cases that PV detectability decreased throughout the gastrointestinal tract, however, traces were still detected after at least 210 min (Figure 3A,B). Our results confirmed that the gastric and intestinal phase degrade and denature proteins, which results in a lower affinity with the antibody [49]. Additionally, Supplementary Materials S3 showed that PV bands are degraded by digestion in the gastric phase. These results confirm the ones obtained by the sandwich ELISA. Significant differences in the gastric phase were determined using a one-way repeated measures ANOVA followed by a paired *t*-test with Bonferroni correction to control a type I error when using multiple *t*-tests. European seabass PV, exposed to pH 3 and 1000:1 pepsin (U) to protein ratio (mg), decreased significantly after 5 min, except for 30 min which showed higher biological variation. Gilthead seabream showed a visible decrease in PV over time, but statistics only showed differences for the first minutes and after half an hour, this might be explained by a high biological variance at 0 min. After two hours of gastric phase, pancreatin and bile salts were added together with the intestinal fluid to the remaining solution. No significant differences were shown during this phase, for both species. A study performed on the stability of cod PV to gastrointestinal conditions showed a low stability after 1 min when subjected to a pH of 1.2, at 0.1 U of pepsin [43].

### 3.5. Fish Processing and Conservation

**Figure 4:**
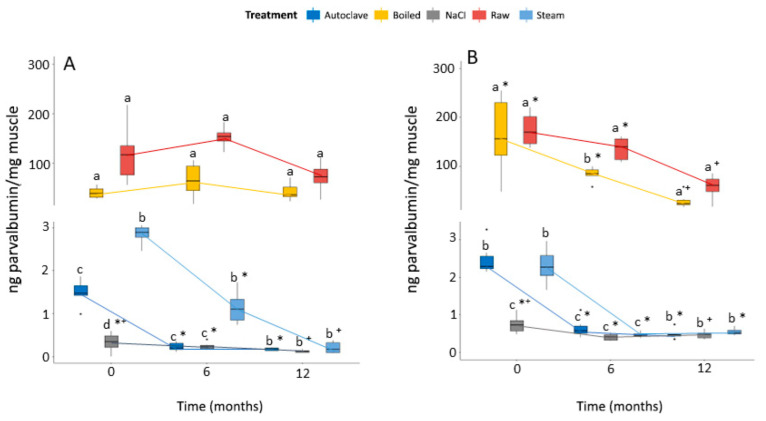
Box plots of European seabass (**A**) and gilthead seabream (**B**) parvalbumin conservation over time (t = 0, 6 and 12 months), with different treatments (Autoclave, Boiled, NaCl, Raw and Steam). Results are shown by quartiles (from Q1 to Q3) and the horizontal line in each box represents the median value. Lines outside the boxes represent the minimum and maximum. Significant differences (two-way repeated measures ANOVA, followed by paired *t*-test with Bonferroni correction) are shown by letters (a, b, c, d) in between treatments at each time point and by symbols (*,^+^) in between time for each treatment. *X*-axis represents time (months) and *y*-axis the ng parvalbumin measured per mg of muscle.

Besides digestion, other methods might also affect PV stability, such as thermal procedures, which are one of the most commonly used techniques in fish processing. They include cooking, steaming and ultra-high heating, which might be accomplished by autoclaving [50]. We used the mentioned thermal processes as follows: boiling fish muscle for 10 min at 98 °C and steaming fish muscle for 8 min at 98–100 °C with or without combination of pressure by autoclaving. Besides the thermal procedures, several non-thermal processes might be used to extend shelf live and enhance the quality [51]. Well-known non-thermal techniques include freezing [18] and salting, which we used as follows: muscle samples were frozen at −20 °C for 12 months and 5% NaCl was added before autoclaving, respectively. Dehydration by freezing seems to be responsible for the exposure of hydrophobic groups, which might result in structural polymers [52]. After each thermal or non-thermal procedure total protein was extracted from the muscle sample, PV was detected using the produced sandwich ELISA with specific antibodies (Figure 4).

At timepoint 0, the thermal process of heating/boiling decreased (slightly) PV detectability in comparison to raw extracts but without significant differences, for both fish species. These results were confirmed in a study with 19 fish species where a positive band for PV was shown after 15 min at 95 °C [53]. Another study also confirmed the heat stable PV presence in 19 out of 22 fish species (except for swordfish, bigeye and yellowfin tuna) after 10 min heating at 100 °C [54]. It should be noted that, although circular dichroism did show a random conformation of parvalbumin when heated to 95–100 °C, it seems that the affinity for the antibodies is still intact. In case of steaming and ultra-high heating using an autoclave (with pressure just over 1 bar), PV decreased significantly in comparison to raw samples, in both species. Studies on proteins showed that pressure can affect their secondary structure [26]. This process is used for fish canning, where elevated temperatures are used followed by packing [18]. Canned tuna is an example of a processed fish species which showed a denatured PV with blocked or destroyed epitopes [27]. Non-thermal processing techniques might also denature proteins, altering their structure as described for thermal processes [18]. Salting, in addition, may affect protein solubilization, resulting in aggregation or precipitation [51]. Addition of NaCl to muscle samples resulted in an even more accentuated decrease in PV content compared to raw samples, in both species used in this study. An SDS-PAGE study performed on salted herring showed a less intense band of PV [27]. In Supplementary Materials S4, an SDS-PAGE performed with all samples showed positive bands for PV, although with less intensity for autoclave, NaCl, and steamed samples, which is in accordance with our sandwich ELISA results. Additionally, it showed that the used processing techniques mainly change the structure of PV and consequently affect the epitopes. Additionally, as observed in Figure 4A, for European seabass, boiled and raw samples showed no further decrease by freezing muscle at −20 °C for 12 months. For gilthead seabream (Figure 4B), we observed a significant reduction in PV detectability after boiling when muscle was frozen for 6 months, in comparison to raw muscle. Additionally, PV from this species reduced its detectability over time with significantly lower amounts after 12 months. Moreover, freezing contributes to an even further decrease in PV after steaming, with significant differences after 6 months at −20 °C for both species, and European seabass showed an even lower amount of PV after 12 months of freezing. Additionally, freezing up to 12 months showed significant differences in PV after autoclaving and salting, comparing to 6 months of being at −20 °C. Freezing, in general, affects protein denaturation, and it seems that storage time influences the level of PV denaturation.

## 4. Conclusions

Our study about the characterization of muscle parvalbumin (PV) from two important southern European fish species, European seabass and gilthead seabream, showed that this allergen has a higher content of α-helices than β-sheets. PV was not completely able to refold back to its original structure, after heating, due to the loss of some α-helices. In vitro digestion was able to show that PV detectability in both species decreased during digestion, suggesting that the affinity of PV antibodies decreased due to structural changes or proteolytic denaturation. Different literature showed the same results with several fish species, although PV detection was performed with other techniques [43,55]. Different processing methods decreased the PV amount, nevertheless, variations between methods showed that salting resulted in the lowest PV detectability. Freezing over an extended time (up to 12 months) decreased PV in almost all studied processing methods, except for boiling in European seabass. The available literature on fish processing using sandwich ELISA (against mackerel PV) only used heated samples, where they did show modifications in PV after pressure cooking [56]. This study contributed to the characterization and stability of PV, a highly allergenic protein in fish. It is important for fish consumers to have this knowledge and be able to evaluate if consumption will be safe in case of a known allergy. Nevertheless, further studies should be performed to analyze IgE-reactivity to fish muscle after being processed.

## Supplementary Materials

The following supporting information can be downloaded at: https://www.mdpi.com/article/10.3390/ani12213022/s1, Figure S1: Parvalbumin detection after heating samples to 90 °C for 10 min; Figure S2: SYPRO ruby stained SDS-PAGE gels from several fractions after gel filtration; Figure S3: SDS-PAGE gels with 15% polyacrylamide from the gastric phase of the in vitro simulation of the gastrointestinal tract of muscle proteins (Coomassie staining); Figure S4: SDS-PAGE with 15% polyacrylamide of samples from several processing techniques without freezing (T0), and after 6 or 12 months of freezing at −20 °C (T6 and T12, respectively).
