# Supplementary material for: Fish Processing and Digestion Affect Parvalbumins Detectability in Gilthead Seabream and European Seabass
Source: Animals (Basel). 2022 Nov 3;12(21):3022. doi: 10.3390/ani12213022 (PMC9654892; doi:10.3390/ani12213022)
Supplement: Supplementary file 1 [file animals-12-03022-s001.zip › animals-1993733-supplementary.pdf]

**Supplemental material and methods (SMM1) –**

Immunization scheme: To produce anti-PV, purified protein was sent to Eurogentec, Belgium. Two rabbits were immunized with purified protein from gilthead seabream and another two rabbits were inoculated with purified protein from European seabass. Antibodies were produced following the classical 87-day program which consists of 4 injections and 4 bleeds. This program produces polyclonal antibodies with high affinities. At the end of the program a protein G purification was performed, by the company.

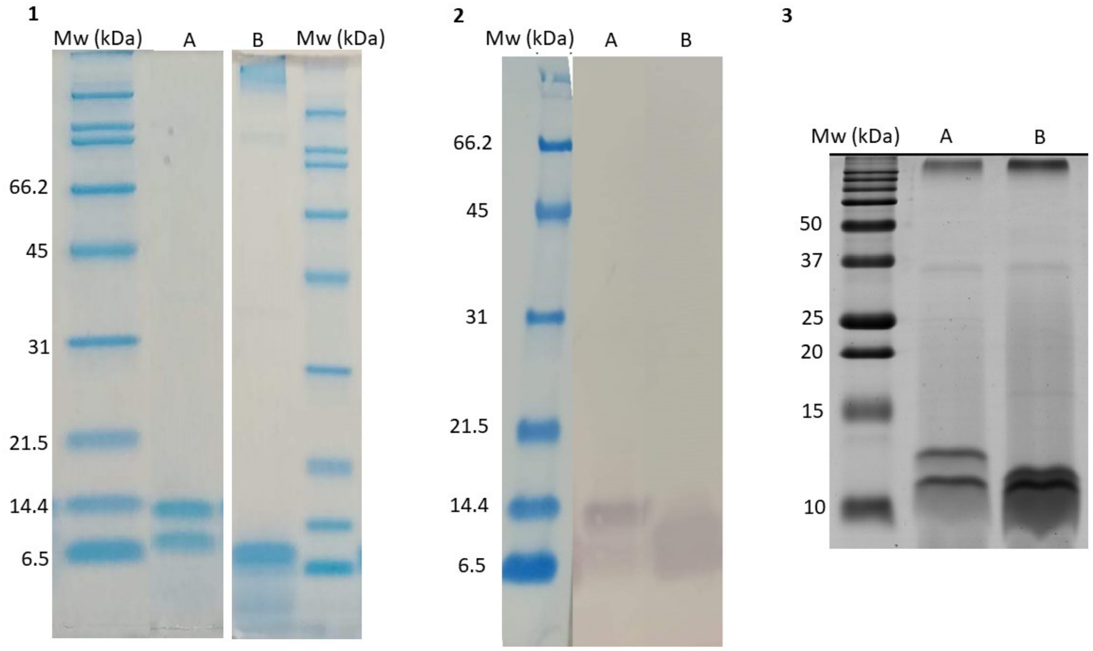


**Figure S1.** Parvalbumin detection after heating samples to 90 °C for 10 min, 1) AnykDa™ Criterion™ TGX™ Precast SDS-PAGE (Bio-rad) with Coomassie staining, 2) Western blot using commercial antibodies (monoclonal anti-parvalbumin, Swant PV235 and anti-rabbit/mouse IgG conjugated with alkaline phosphatase, Sigma, 1:10,000 diluted in blocking buffer) and 3) 18% SDS-PAGE gels with Coomassie staining. Mw—Molecular weight (kDa). A—European seabass. B—gilthead seabream.

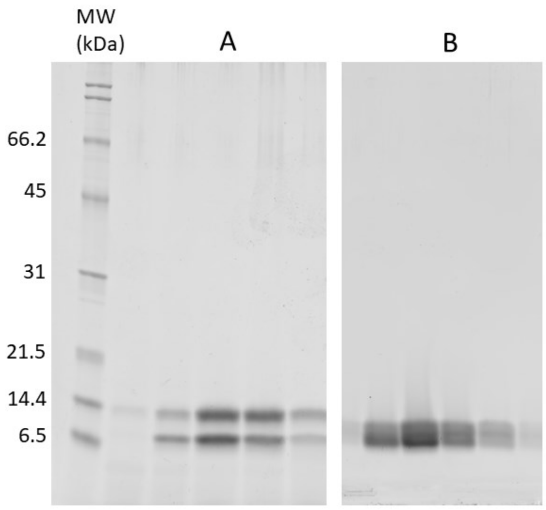


**Figure S2.** SYPRO ruby stained SDS-PAGE gels from several fractions after gel filtration. Mw—Molecular weight (kDa). A—European seabass and B—gilthead seabream.

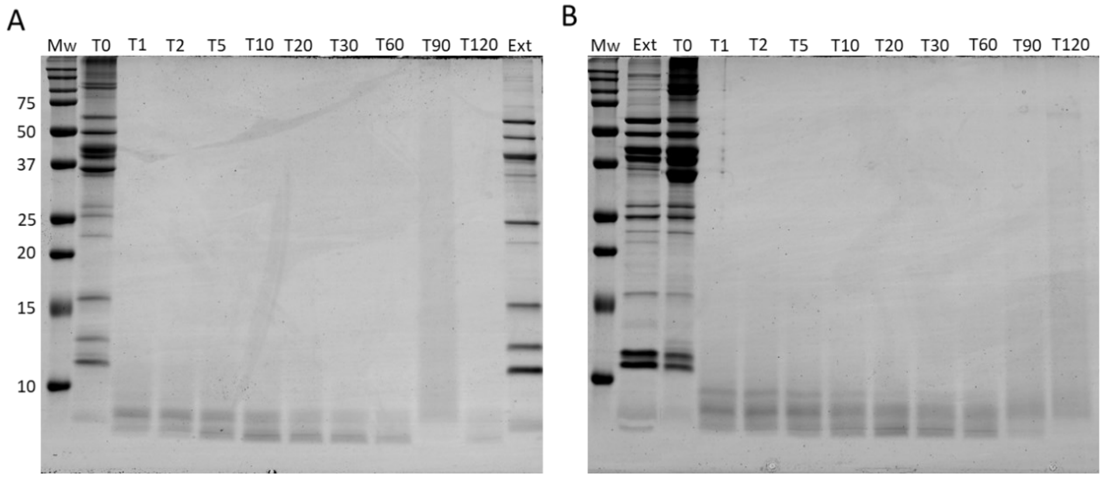


**Figure S3.** SDS-PAGE gels with 15% polyacrylamide from the gastric phase of the in vitro simulation of the gastrointestinal tract of muscle proteins (Coomassie staining). Mw—molecular weight (kDa). A—European seabass and B—gilthead seabream. Ext—crude extract.

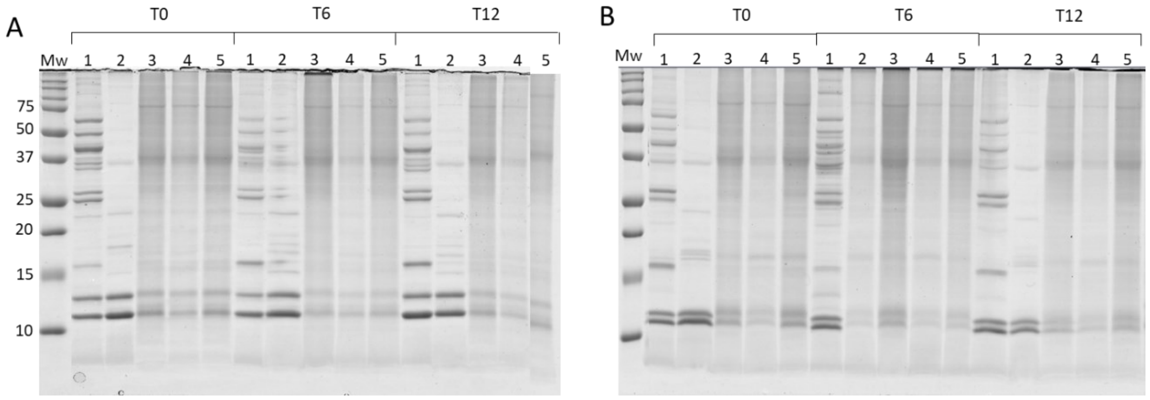


**Figure S4.** SDS-PAGE with 15% polyacrylamide of samples from several processing techniques without freezing (T0), and after 6 or 12 months of freezing at −20 °C (T6 and T12, respectively). A—European seabass and B—gilthead seabream. 1—Raw, 2—Boiled, 3—Autoclave, 4—NaCl, 5—Steam.
